# How to make hand sanitiser gel

**Published:** 2020-09-01

**Authors:** Choon Fu Goh, Abeer HA Mohamed Ahmed

**Affiliations:** 1 Senior Lecturer in Pharmaceutical Technology/ Leading Researcher in Skin Research Group: School of Pharmaceutical Sciences, Universiti Sains Malaysia, Minden, Penang, Malaysia.; 2 Research Fellow in Pharmacology and Clinical Trials, Pharmacist: London School of Hygiene & Tropical Medicine, London, UK.

**Alcohol-based hand sanitisers can be made in the form of a gel, which allows the alcohol to remain in contact for the hands for longer while hand washing/hand rubbing.**

This hand sanitiser gel is suitable for use by the public and can be prepared on a small scale at home or in a laboratory, using basic equipment. For the hand gel to be effective against viruses such as SARS-CoV-2, the final product should contain 80% ethanol **or** 75% isopropyl alcohol. Ethanol is less concentrated than isopropyl alcohol, so you will need to use slightly more.

The World Health Organization recommends that hand sanitisers are used on skin with **no visible dirt**. If your hands are visibly dirty, wash them with soap and water.

## What you will need

To make 100 ml (millilitres) of hand gel, you will need:

### Ingredients

- Alcohol: ethanol 96% (83.3 ml) or isopropyl alcohol 99.8% (75.2 ml)
- Emollient: glycerol/glycerine/glycerin 98% (4 ml)
- Gelling agent: hydroxypropyl methylcellulose (1 g)
- Sterile distilled or cooled boiled water (20 ml)

### Equipment

- A beaker or other container for mixing (250 ml)
- Measuring cylinders and measuring jugs
- Micropipette (optional)
- Plastic or silicone spatula
- Weighing balance or electronic scales
- Electric mixer or homogeniser
- A glass or plastic dispensing bottle (minimum 100 ml in volume)

## Procedure

**Figure 1:**
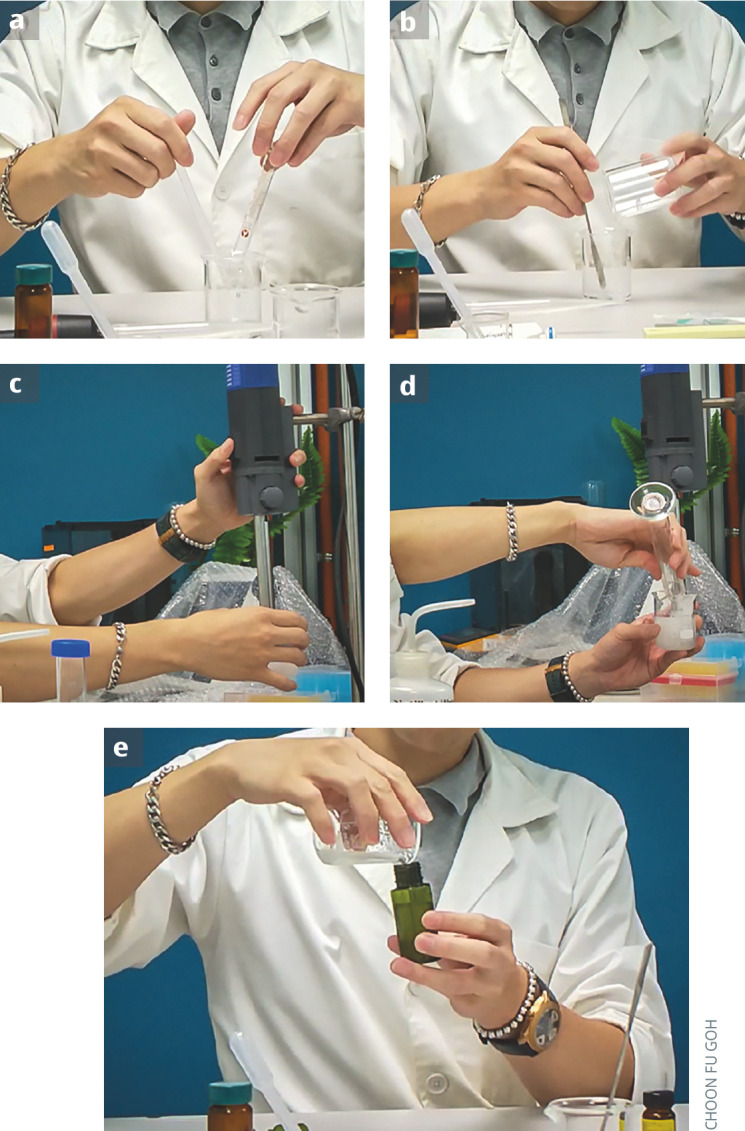
Preparation of alcohol-based hand sanitiser gel

- Clean the working surfaces.
- Wash your hands and put on a clean lab coat or an apron.
- Gather the ingredients and place within easy reach
- Graduate the final dispensing bottle by marking the level equivalent to 100 ml.
- Measure the alcohol (83.3 ml of ethanol or 75.2 ml of isopropyl alcohol) using a measuring jug or cylinder.
- Cover (to avoid evaporation) and set aside.
- Measure 20 ml of sterile distilled water or boiled cold water using a measuring jug or cylinder. Set aside until needed.
- Measure 4 ml of glycerol using a measuring cylinder or micropipette and then pour it into the beaker or container (Figure 1a). Rinse the measuring cylinder with some of the sterile distilled water or cooled boiled water to remove the remaining glycerol and pour it into the mixing container.
- Weigh 1 g of hydroxypropyl methylcellulose and disperse it gradually into the solution mixture to avoid the formation of gel lumps. Use a mixer or homogeniser to hasten the process (Figure 1b and 1c).
- Add the measured alcohol into the mixing container (Figure 1d). A mixer or homogeniser can be used to help the mixing process.
- Empty the solution into the dispensing bottle (Figure 1e). Use a plastic or silicone spatula if needed.
- Add sterile distilled water or cold boiled water into the dispensing bottle, up to the 100 ml mark.
- Place the lid on the dispensing bottle as soon as possible to prevent evaporation.
- Mix the solution by shaking the bottle gently.
- Store the solution for 72 hours before use to make sure that any microbes in the alcohol or the bottles will be destroyed.
- Label the bottle and list the final concentrations of ingredients (see Table 1).

**Table 1:** Final concentrations of ingredients in hand sanitiser gel

Hand sanitiser gel
Ethanol 80% (or isopropyl alcohol 75%) Glycerol 4% Hydroxypropyl methylcellulose 1% Water 15% or 20% depending on type of alcohol used
